# Integrative analysis identifies AKAP8L as an immunological and prognostic biomarker of pan-cancer

**DOI:** 10.18632/aging.205003

**Published:** 2023-09-07

**Authors:** Libo Zhou, Jinhong Mei, Runfu Cao, Xiaoqiang Liu, Bin Fu, Ming Ma, Binbin Gong, Lianmin Luo, Yifu Liu, Qiqi Zhu, Xuan Meng

**Affiliations:** 1 Department of Urology, The First Affiliated Hospital of Nanchang University, Nanchang 330006, Jiangxi, P.R. China; 2 Department of Pathology, The First Affiliated Hospital of Nanchang University, Nanchang 330006, Jiangxi, P.R. China

**Keywords:** AKAP8L, pan-cancer analysis, prognosis, biomarker, bioinformatics analysis

## Abstract

A-kinase anchoring protein 8L (AKAP8L) belong to the A-kinase anchoring protein (AKAP) family. Recent studies have proved that AKAP8L is associated with the progression of various tumors. To establish a more complete understanding of the significance of AKAP8L across various types of cancers, we conducted a detailed analysis of multiple histological datasets, including the level of gene expression in pancancer, biological function, molecular characteristics, as well as the diagnostic and prognostic value of AKAP8L in pancancer. Furthermore, we focused on renal clear cell carcinoma (KIRC), and of explored the correlation of AKAP8L with clinical characteristics, prognosis of distinct patient subsets, co-expression genes and differentially expressed genes (DEG). We also performed the immunohistochemical staining and semi-quantitative verification of the monoclonal antibody established by AKAP8L. Our findings indicate that AKAP8L expression varied significantly not only across most cancer types, but also across different cancer molecules and immune subtypes. In addition, the robust ability to accurately predict cancer and its strong correlation with the prognosis of cancer strongly suggest that AKAP8L may be a potential biomarker for cancer diagnosis and prognosis. Furthermore, the high expression levels of AKAP8L were related to the worse overall survival (OS), disease-specific survival (DSS) as well as progression-free interval (PFI) of KIRC with statistical significance, especially among distinct clinical subgroups of KIRC. To sum up, AKAP8L has the potential to serve as a critical molecular biomarker for the diagnosis and prognosis of pancancer, an independent prognostic risk factor of KIRC, and a novel molecular target for cancer therapies.

## INTRODUCTION

A-kinase anchoring proteins (AKAPs) are a family of proteins with different structures and related functions [1]. Their main function is to anchor cyclic adenosine monophosphate (cAMP)-dependent protein kinase A to specific subcellular structures [2]. In addition, AKAPs play a key role in creating spatial boundaries for the combination of multiple signals. AKAPs serve as an organizing center for various protein kinases and phosphatases in G protein-coupled receptors, creating a signal device that can send signals, regulate and transport within cells [1, 3]. Therefore, AKAPs involve many biological processes [4–6]. Recently, studies have verified that AKAPs may regulate the proliferation, invasion as well as survival of tumor cells and play different roles in different cancers [7, 8].

With the gradual deepening of the research on AKAPs gene, the functions of AKAP family members have garnered attention from many researchers. AKAP8 belongs to the AKAP family and participates in a variety of biological processes [9]. Some studies have proved that the zinc finger domain (ZF) of AKAP8 could combine with lots of factors in RNA processing / transcription to regulate biological processes such as transcription and RNA splicing [10, 11]. Sho et al. demonstrated that AKAP8 participated in the regulation of chromatin structure changes through nuclear tyrosine phosphorylation [12]. Recent studies have confirmed that AKAP8 could suppress the metastasis of tumors via adjusting the splicing isomer of CLSTN1, a molecule that controls epithelial mesenchymal transition [13]. Despite of that, Li et al. confirmed that AKAP8 might promote tumorigenesis by forming a liquid condensate in the nucleus of cancer and modulating selective splicing [14].

The protein sequence of AKAP8L exhibits a similarity of up to 61% with that of AKAP8, suggesting that AKAP8L and AKAP8 may have similar functions in tumor genesis and metastasis. It has been previously proved that the high AKAP8L expression is associated with poor prognosis in esophageal squamous cell carcinoma, serving as an independent prognostic factor for the disease [9]. Zhang et al. found that AKAP8L can enhance the dryness and chemoresistance of gastric cancer cells by stabilizing the expression of SCD1 mRNA [15]. A previous study proved that AKAP8L can promote cell growth by interacting with mTORC1 [16].

As previously reported, AKAP8L may play a part in different cancers. Currently, there has been limited research on the role of AKAP8L in various types of malignancies. Thus, its function in most types of cancers remains unclear. To gain a comprehensive understanding of AKAP8L, we explored its expression as well as biological function of AKAP8L across various types of cancer, with a focus on its potential as a marker for early diagnosis and outcome prediction. We found that AKAP8L was up-regulated or down-regulated in 14 human cancers, and its expression varied across molecular subtypes of 11 cancer types as well as immune subtypes of 7 cancer types. Furthermore, AKAP8L exhibited a strong predictive value for the identification of testicular germ cell tumors (TGCT), liver hepatocellular carcinoma (LIHC), rectum adenocarcinoma (READ) and colon adenocarcinoma (COAD), and has significant correlation with the total survival rate (OS), disease-specific survival rate (DSS) and progression-free interval (PFI) of COAD, kidney renal clear cell carcinoma (KIRC), kidney renal papillary cell carcinoma (KIRP), as well as prostate adenocarcinoma (PRAD). Then, we laid an emphasis on KIRC and determine AKAP8L as the independent risk factor of OS, DSS as well as PFI in uterine corpus endometrial cancer (UCEC). Besides, we conducted further analyses on the co-expression genes related to AKAP8L, as well as the differentially expressed genes (DEG) between the high and low expression groups of AKAP8L. In conclusion, AKAP8L has the potential to serve as a valuable biomarker for the early diagnosis and outcome prediction of pancancer and may represent a prospective molecular target of KIRC.

## RESULTS

### AKAP8L expression in pan-cancer

**Figure 1:**
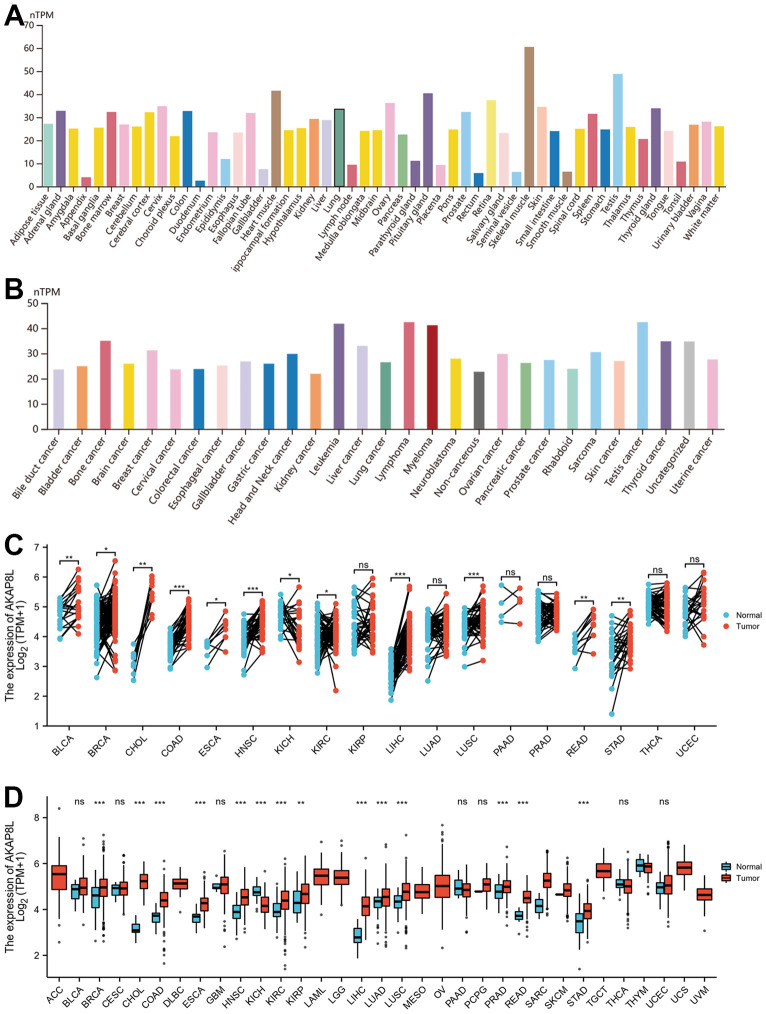
**Expression level of AKAP8L gene in tumors and normal tissues.** (**A**) AKAP8L expression in normal tissues; (**B**) AKAP8L expression in tumor cell lines; (**C**) AKAP8L expression in TCGA tumors and adjacent normal tissues; (**D**) AKAP8L expression in TCGA tumors and normal tissues (*p < 0.05, **p < 0.01, ***p < 0.001).

HPA database was adopted to analyze AKAP8L expression within normal tissues, which revealed the expression of AKAP8L in the majority of normal tissues, with the highest in the skeletal muscle (Figure 1A). Similarly, AKAP8L was also expressed among most tumor cell lines (Figure 1B). Compared to adjacent normal tissues using TCGA datasets, a significant up-regulation of AKAP8L expression was observed in 12 cancer types, such as bladder urothelial carcinoma (BLCA), breast invasive carcinoma (BRCA), cholangiocarcinoma (CHOL), COAD, esophageal carcinoma (ESCA), head and neck squamous cell carcinoma (HNSC), renal chromophobe (HNSC), KIRC, LIHC, lung squamous cell carcinoma (LUSC), READ, and stomach adenocarcinoma (STAD) (Figure 1C). In addition, compared to non-adjacent normal tissues using TCGA datasets, a significant up-regulation of AKAP8L expression was observed in 14 cancer types, such as BRCA, CHOL, COAD, ESCA, HNSC, KICH, KIRC, kidney renal papillary cell carcinoma (KIRP), LIHC, Lung adenocarcinoma (LUAD), LUSC, Prostate adenocarcinoma (PRAD), READ, as well as STAD (Figure 1D).

### Association of AKAP8L with molecular or immune subtypes of cancers/carcinoma

TISDB database was adopted to analyze the association of AKAP8L expression with molecular subtypes across various types of cancer, and our results indicated that AKAP8L expression was significantly varied among distinct molecular subtypes of 11 cancer types (UCEC, BRCA, LUSC, OV, LGG, STAD, KIRP, ACC, COAD and glioblastoma multiforme [GBM]).

**Figure 2:**
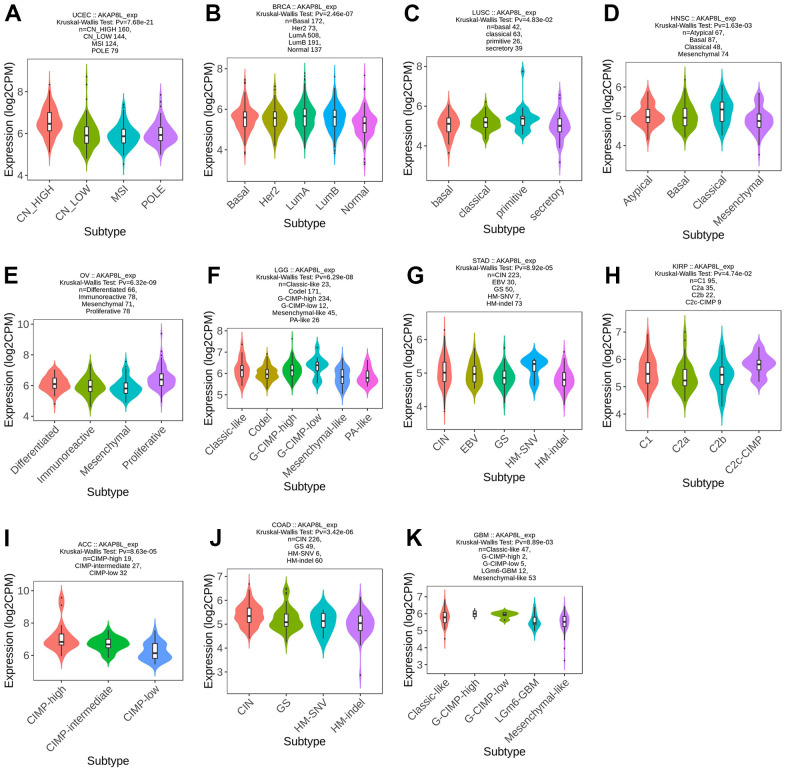
**Correlations between AKAP8L expression and molecular subtypes across TCGA tumors.** (**A**) UCEC; (**B**) BRCA; (**C**) LUSC; (**D**) HNSC; (**E**) OV; (**F**) LGG; (**G**) STAD; (**H**) KIRP; (**I**) ACC; (**J**) COAD; (**K**) GBM.

In addition, AKAP8L expression was the highest in the subtypes of CN_ HIGH, LumA, primitive molecular subtypes, classical molecular subtypes, proliferative subtypes, G-CIMP-low subtypes, CIN molecular subtypes, C2a molecular subtypes, CIMP-high subtypes, CIN molecular subtypes, and Classic-like subtypes (Figure 2A–2K) for UCEC (P=7.68e-21), BRCA (P=2.46e-07), LUSC (P=4.83e-02), HNSC (P=1.63e-03), OV (P=6.32e-09), LGG (P=6.29e-08), STAD (P=8.92e-05), KIRP (P=4.74e-02), ACC (P=8.63e-05), COAD (P=3.42e-06), and GBM (P=8.89e-03), respectively.

**Figure 3:**
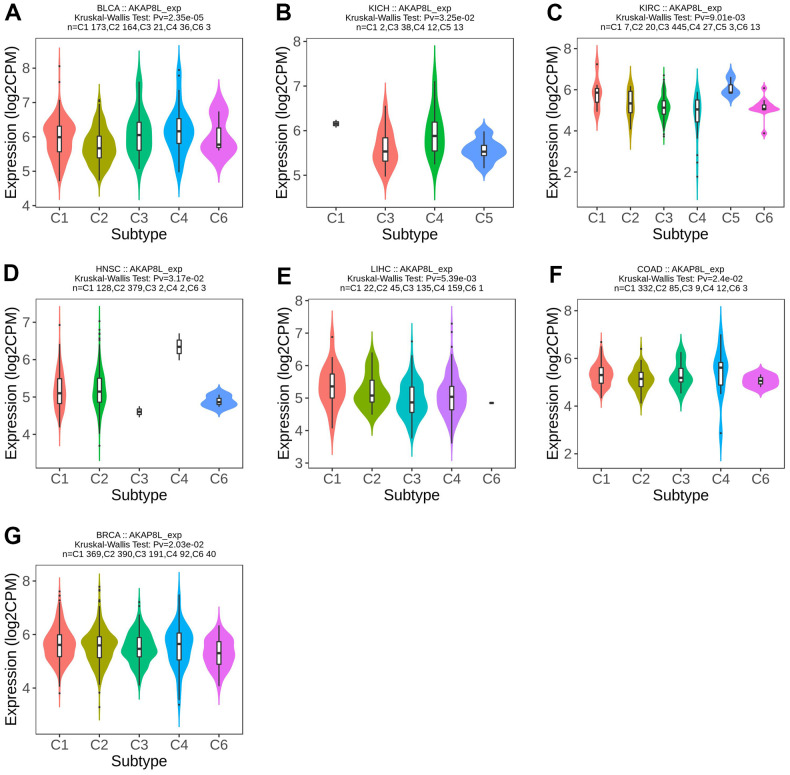
**Correlations between AKAP8L expression and immune subtypes across TCGA tumors.** (**A**) BLCA; (**B**) KICH; (**C**) KIRC; (**D**) HNSC; (**E**) LICH; (**F**) COAD; (**G**) BRCA.

Meanwhile, AKAP8L expression was found to be related to different immune subtypes (C1: wound healing, C2: IFN- γ Dominant, C3: inflammation, C4: lymphocyte depletion, C5: immune silence, C6: TGF-b dominance) of 7 tumor types, including BLCA (P=2.35e-05), KICH (P=3.25e-02), KIRC (P=9.01e-03), HNSC (P=3.17e-02), LICH (P=5.39e-03), COAD (P=2.4e-02), and BRCA (P=2.03e-02) (Figure 3A–3G).

### AKAP8L promoter methylation level in pan-cancer

**Figure 4:**
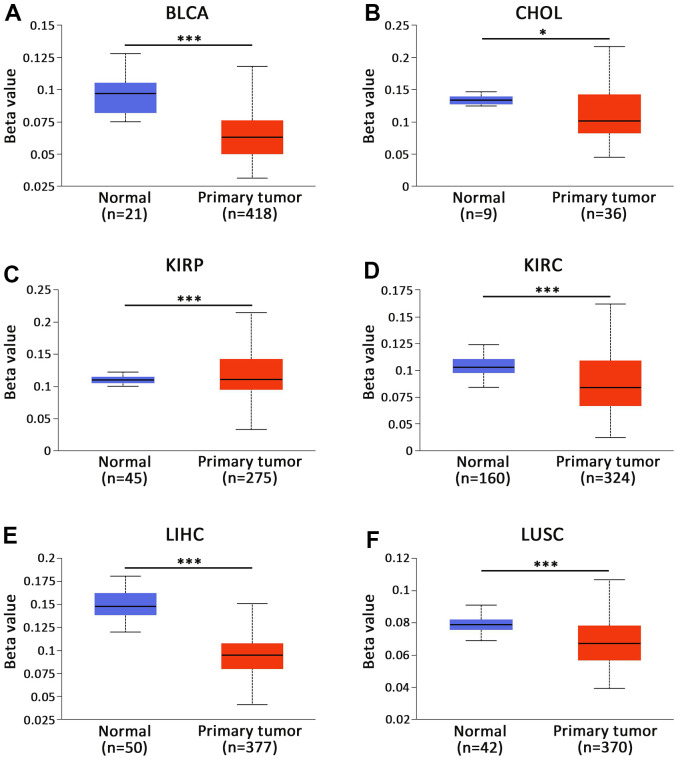
**Promoter methylation status of CBXs in BLCA (UALCAN).** The promoter of AKAP8L was hypomethylated in (**A**) BLCA and (**B**) CHOL tissues. The promoter of AKAP8L was hypermethylated in (**C**) KIRP tissues. The promoter of AKAP8L was hypomethylated in (**D**) KIRC, (**E**) LIHC and (**F**) LUSC tissues. * *p* < 0.05, *** *p* < 0.001.

UALCAN database was adopted for the assessment of the methylation level of AKAP8L across various types of cancer [17, 18] and found a close relationship between the methylation of AKAP8L promoter and the progression of various tumors, including BLCA, CHOL, KIRP, KIRC, LIHC and LUSC. We found that AKAP8L promoter was hypermethylated in CHOL (Figure 4B), KIRP (Figure 4C), KIRC (Figure 4D) and LIHC (Figure 4E); in contrast, AKAP8L promoter was hypomethylated in BLCA (Figure 4A) as well as LUSC (Figure 4F).

### PPI network and enrichment analysis of GO and KEGG

**Figure 5:**
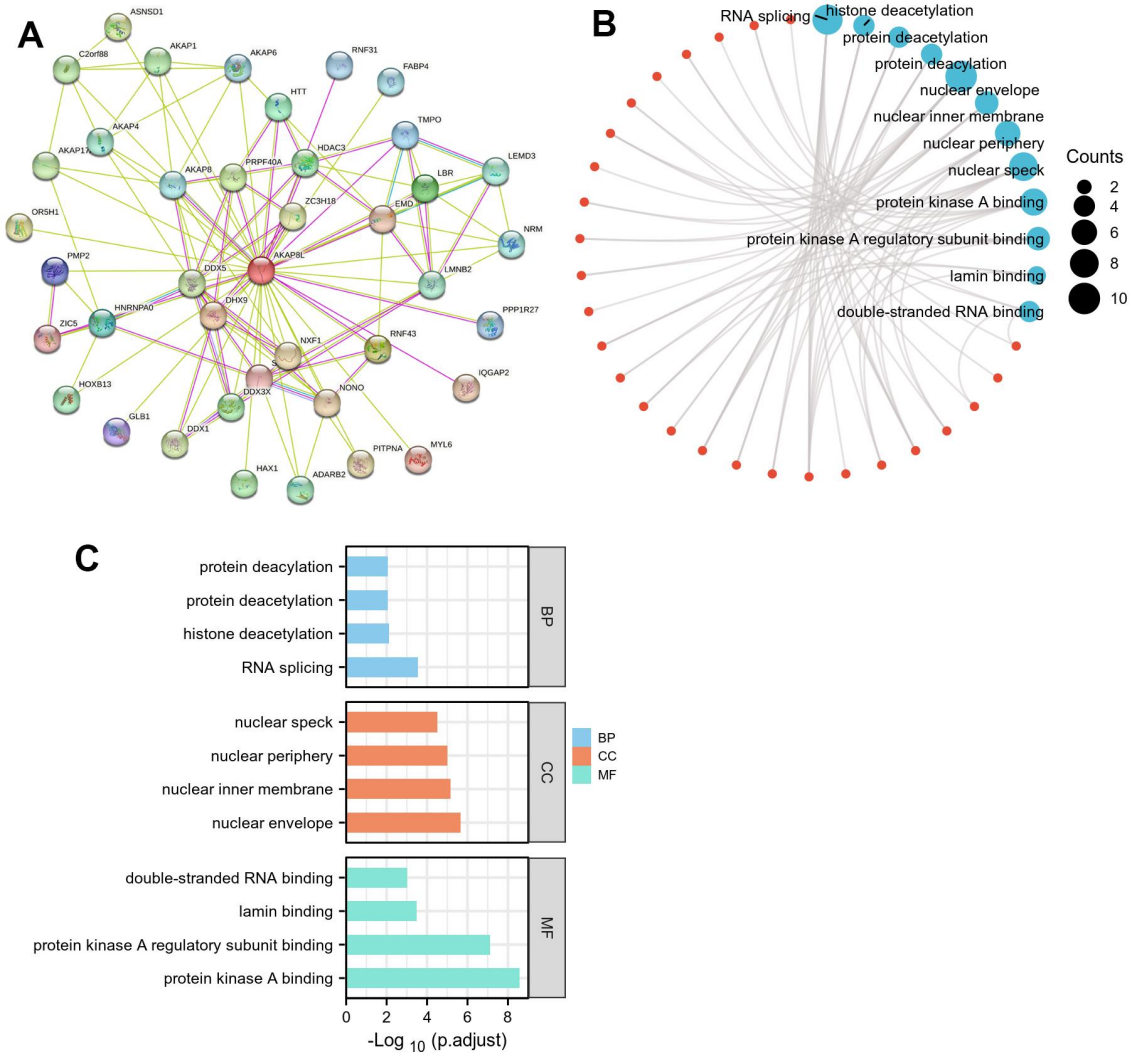
**Protein–protein interaction (PPI) network, GO analysis, and KEGG analysis of 50 targeted binding proteins of AKAP8L.** (**A**) PPI network; (**B**) visual network of GO and KEGG analyses; (**C**) GO analysis.

STRING database was adopted to screen 50 target binding proteins (Figure 5A). Subsequently, we carried out GO enrichment analysis on 50 target binding proteins, demonstrating that the primary biological process (BP) included protein deacetylation, histone deacetylation, along with RNA splicing. Besides, cell components (CC) were mainly enriched in nuclear speck, nuclear periphery, nuclear inner membrane, as well as nuclear envelope. In addition, molecular function (MF) mainly involved double-stranded RNA binding, lamin binding, protein kinase A regulatory subunit binding, together with protein kinase A binding (Figure 5B, 5C). However, results of KEGG pathway enrichment analysis proved that there was no relevant enrichment pathway.

### Diagnostic value of AKAP8L in pan-cancer

**Figure 6:**
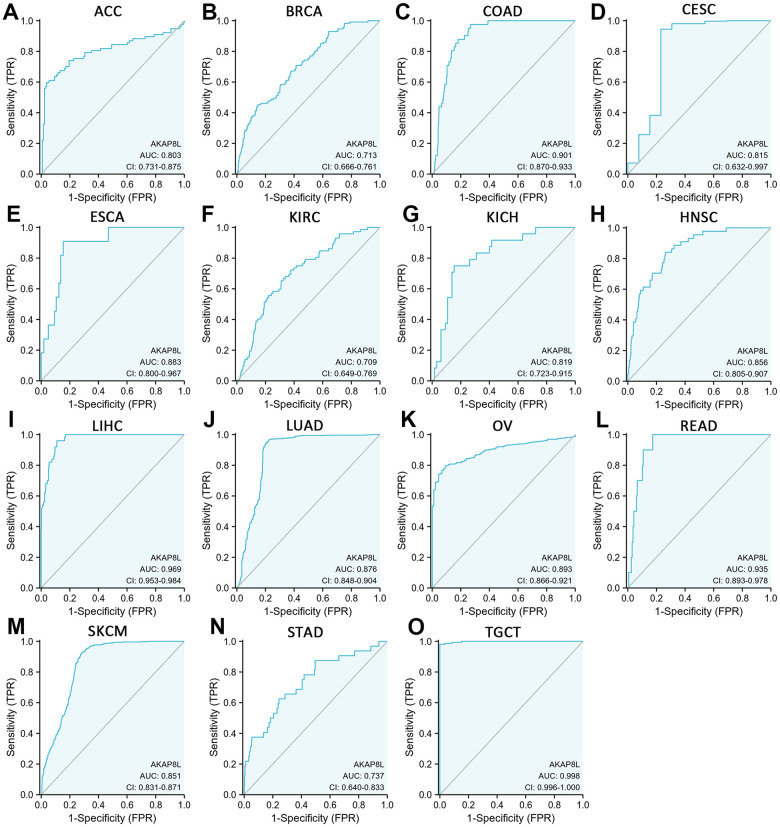
**Receiver operating characteristic (ROC) curve for AKAP8L expression in pan-cancer.** (**A**) ACC; (**B**) BRCA; (**C**) COAD; (**D**) CESC; (**E**) ESCA; (**F**) KIRC; (**G**) KICH; (**H**) HNSC; (**I**) LIHC; (**J**) LUAD; (**K**) OV; (**L**) READ; (**M**) SKCM; (**N**) STAD; (**O**) TGCT.

We employed receiver operating characteristic (ROC) curve to evaluate AKAP8L’s diagnostic ability across various cancer types, and our results indicated that AKAP8L demonstrated a certain level of accuracy in diagnosing 15 cancer types with an area under the curve (AUC) greater than 0.7. The AUCs for these cancer types were as follows: ACC (AUC=0.803), BRCA (AUC=0.713), COAD (AUC=0.901), CESC (AUC=0.815), ESCA (AUC=0.883), KIRC (AUC=0.709), KICH (AUC=0.819), HNSC (AUC=0.855), LIHC (AUC=0.969), LUAD (AUC=0.876), OV (AUC=0.893), READ (AUC=0.935), SKCM (AUC=0.851), STAD (AUC=0.737) and TGCT (AUC=0.998) (Figure 6A–6O).

### Prognostic value of AKAP8L for cancers

**Figure 7:**
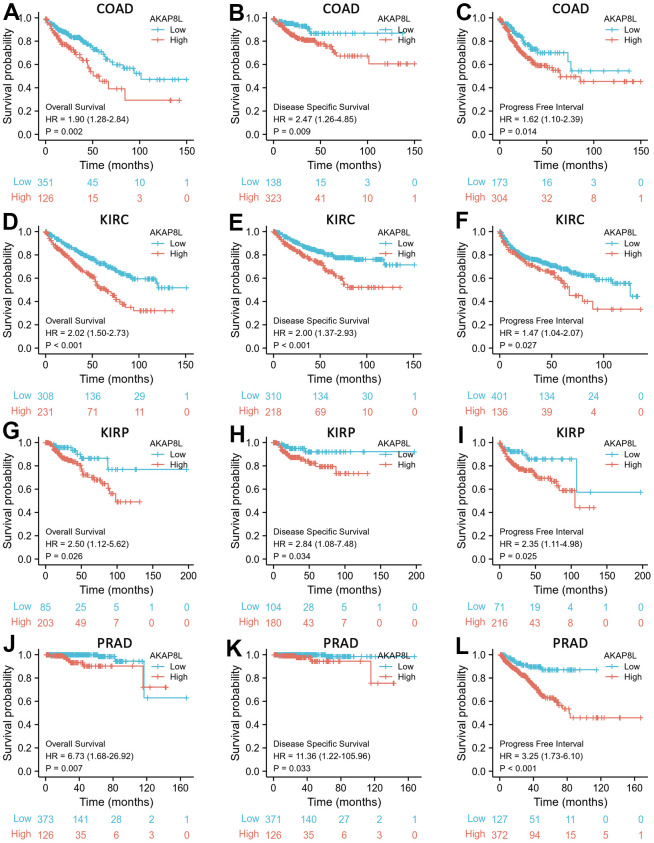
**Correlations between AKAP8L expression and the prognosis (OS, DSS, and PFI) of cancers.** (**A**–**C**) COAD; (**D**–**F**) KIRC; (**G**–**I**) KIRP; (**J**–**L**) PRAD.

Based on cox regression analysis, we observed that a higher expression of AKAP8L was significantly associated with a worse prognosis in COAD, KIRC, KIRP, and PRAD. Specifically, the prognostic markers included OS [hazard ratio (HR)=1.90, 95% confidence interval (CI): 1.28 – 2.84, p=0.002], DSS (HR=2.47, 95% CI: 1.26 – 4.85, p=0.009), PFI (HR=1.62, 95% CI=1.10 – 2.39, p=0.014) for COAD (Figure 7A–7C); OS (HR=2.02, 95% CI: 1.50-2.73, p<0.001), DSS (HR=2.00, 95% CI: 1.37-2.93, p<0.001) and PFI (HR=1.47, 95% CI: 1.04-2.07, p=0.027) for KIRC (Figure 7D–7F); OS (HR=2.50, 95% CI: 1.12-5.62, p=0.026), DSS (HR=2.84, 95% CI: 1.08-7.48, p=0.034) and PFI (HR=2.35, 95% CI: 1.11-4.98, p=0.025) for KIRP (Figure 7G–7I); Finally, OS (HR=6.73, 95% CI: 1.68-26.92, p=0.007), DSS (HR=11.36, 95% CI: 1.22-105.96, p=0.033) and PFI (HR=3.25, 95% CI: 1.73-6.10, p<0.001) for PRAD (Figure 7J–7L).

**Figure 8:**
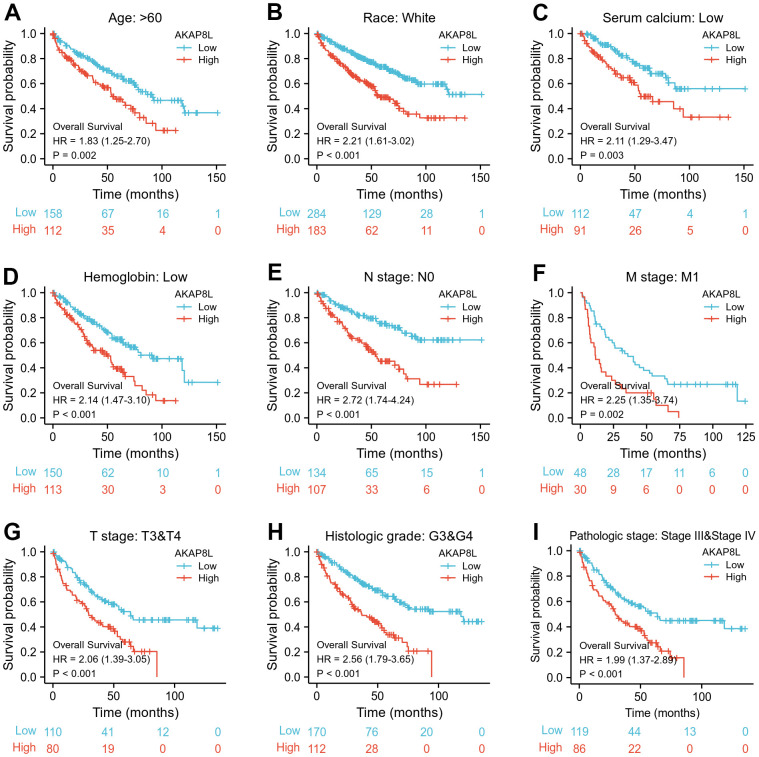
**Associations between AKAP8L expression and the OS in different clinical subgroups of KIRC.** (**A**) Age > 60; (**B**) Race: White; (**C**) Serum calcium: Low; (**D**) Hemoglobin: Low; (**E**) N stage: N0; (**F**) M stage: M1; (**G**) T stage: T3&T4; (**H**) Histologic grade: G3&G4; (**I**) Pathologic stage: Stage III & Stage IV.

**Figure 9:**
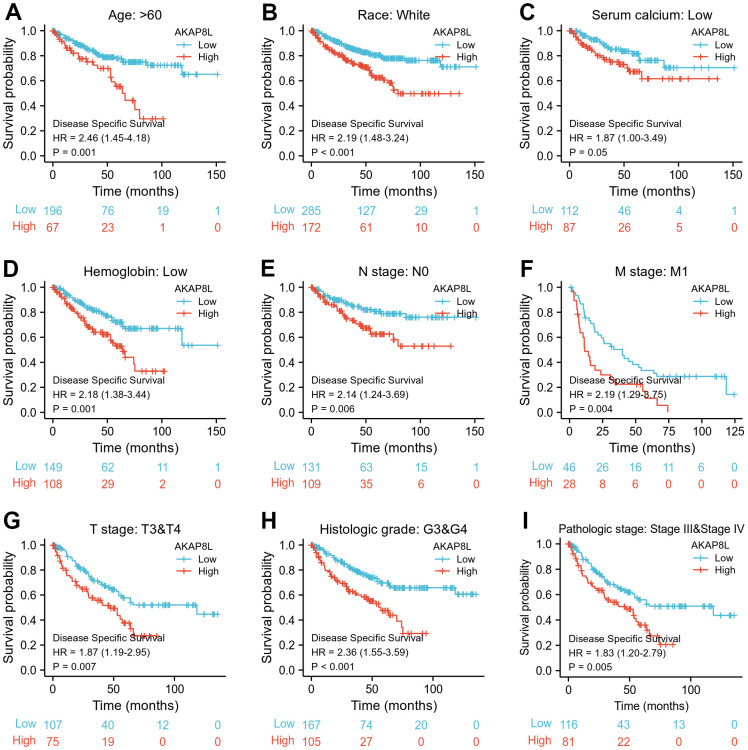
**Associations between AKAP8L expression and the DSS in different clinical subgroups of KIRC.** (**A**) Age > 60; (**B**) Race: White; (**C**) Serum calcium: Low; (**D**) Hemoglobin: Low; (**E**) N stage: N0; (**F**) M stage: M1; (**G**) T stage: T3&T4; (**H**) Histologic grade: G3&G4; (**I**) Pathologic stage: Stage III & Stage IV.

**Figure 10:**
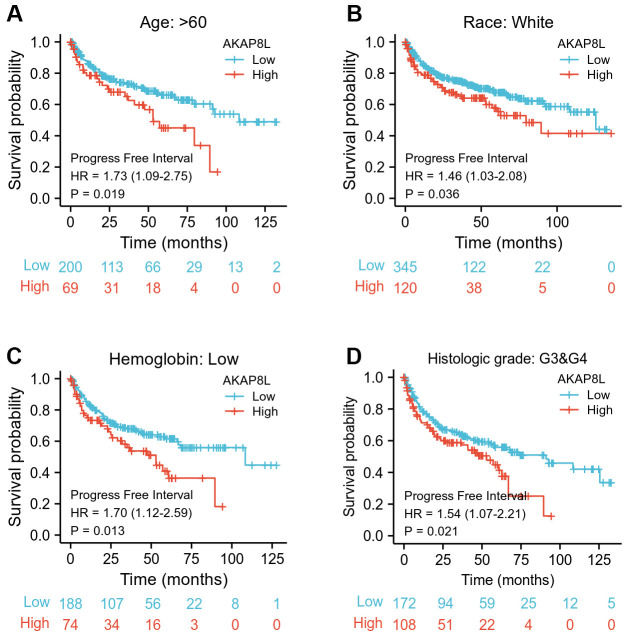
**Associations between AKAP8L expression and the PFI in different clinical subgroups of KIRC.** (**A**) Age > 60; (**B**) Race: White; (**C**) Hemoglobin: Low; (**D**) Histologic grade: G3&G4.

Besides, we investigated the correlation between AKAP8L and the prognosis of distinct clinical subgroups of KIRC (OS, DSS and PFI), and observed a higher AKAP8L expression as well as the worse OS in subgroups with an age > 60, white race, lower serum calcium and hemoglobin, no lymphatic metastasis (N0), distant metastasis (M1), clinical tumor staging of T3-T4, histologically graded G3-G4 and pathologically graded Stage III-IV, as demonstrated in Figure 8A–8H. The relationship of AKAP8L expression with DSS were consistent with that with OS, as shown in Figure 9A–9I. A higher AKAP8L expression was associated with the worse PFI among the subgroups with an age>60, white race, lower hemoglobin and histological grade of G3-G4 (Figure 10A–10D).

### The AKAP8L expression in KIRC

**Figure 11:**
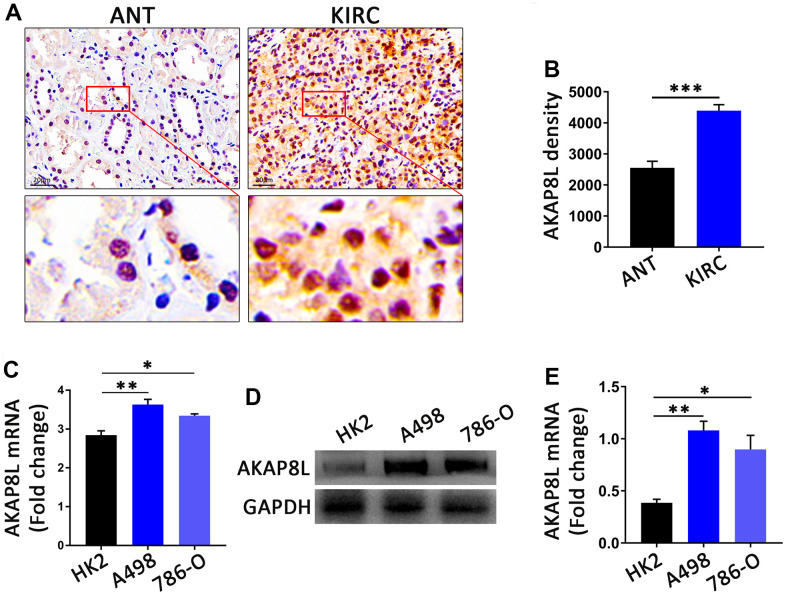
**The AKAP8L expression in KIRC.** (**A**) Representative images showing immunohistochemica (IHC) staining results of AKAP8L in KIRC tissues (KIRC) and adjacent normal tissues (ANT) (scale bar, 20 μm). (**B**) Semiquantitative analysis of IHC staining (n=10). (**C**) The mRNA expression levels of AKAP8L in different cell types (n≥3). (**D**) Protein expression levels of AKAP8L and GAPDH in different cell types. (**E**) Semi-quantification of AKAP8L protein expression level determined by densitometry normalized to GAPDH. ^*^P<0.05, ^**^P<0.01, ^***^P<0.001. KIRC, Kidney renal clear cell carcinoma.

IHC analysis revealed that in all 10 pairs of samples, compared to adjacent normal tissue, AKAP8L protein expression was higher in KIRC tumor tissues with statistical significance (Figure 11A, 11B). Consistent with the aforementioned results, RT-qPCR and Western blot (WB) analysis also showed a higher AKAP8L expression in KIRC (A498 and 786-O) cells compared to normal human kidney (HK2) cells with statistical significance (Figure 11C–11E).

### Relationship of AKAP8L with different clinical characteristics of KIRC

The expression of AKAP8L was associated with the sex, race as well as histological grade of KIRC with statistical significance (Table 1).

**Table 1:** Clinical characteristics of KRIC patients.

**Characteristic**	**Levels**	**Low expression of AKAP8L**	**High expression of AKAP8L**	**p**
n		269	270	
Gender, n (%)	Female	78 (14.5%)	108 (20%)	0.009
	Male	191 (35.4%)	162 (30.1%)	
Race, n (%)	Asian	1 (0.2%)	7 (1.3%)	< 0.001
	Black or African American	16 (3%)	41 (7.7%)	
	White	249 (46.8%)	218 (41%)	
Histologic grade, n (%)	G1	0 (0%)	14 (2.6%)	0.002
	G2	119 (22.4%)	116 (21.8%)	
	G3	107 (20.2%)	100 (18.8%)	
	G4	39 (7.3%)	36 (6.8%)	
Hemoglobin, n (%)	Elevated	2 (0.4%)	3 (0.7%)	0.046
	Low	125 (27.2%)	138 (30.1%)	
	Normal	112 (24.4%)	79 (17.2%)	
OS event, n (%)	Alive	196 (36.4%)	170 (31.5%)	0.018
	Dead	73 (13.5%)	100 (18.6%)	
DSS event, n (%)	Alive	220 (41.7%)	200 (37.9%)	0.026
	Dead	43 (8.1%)	65 (12.3%)	

### Co-expression gene analysis of AKAP8L in KIRC

**Figure 12:**
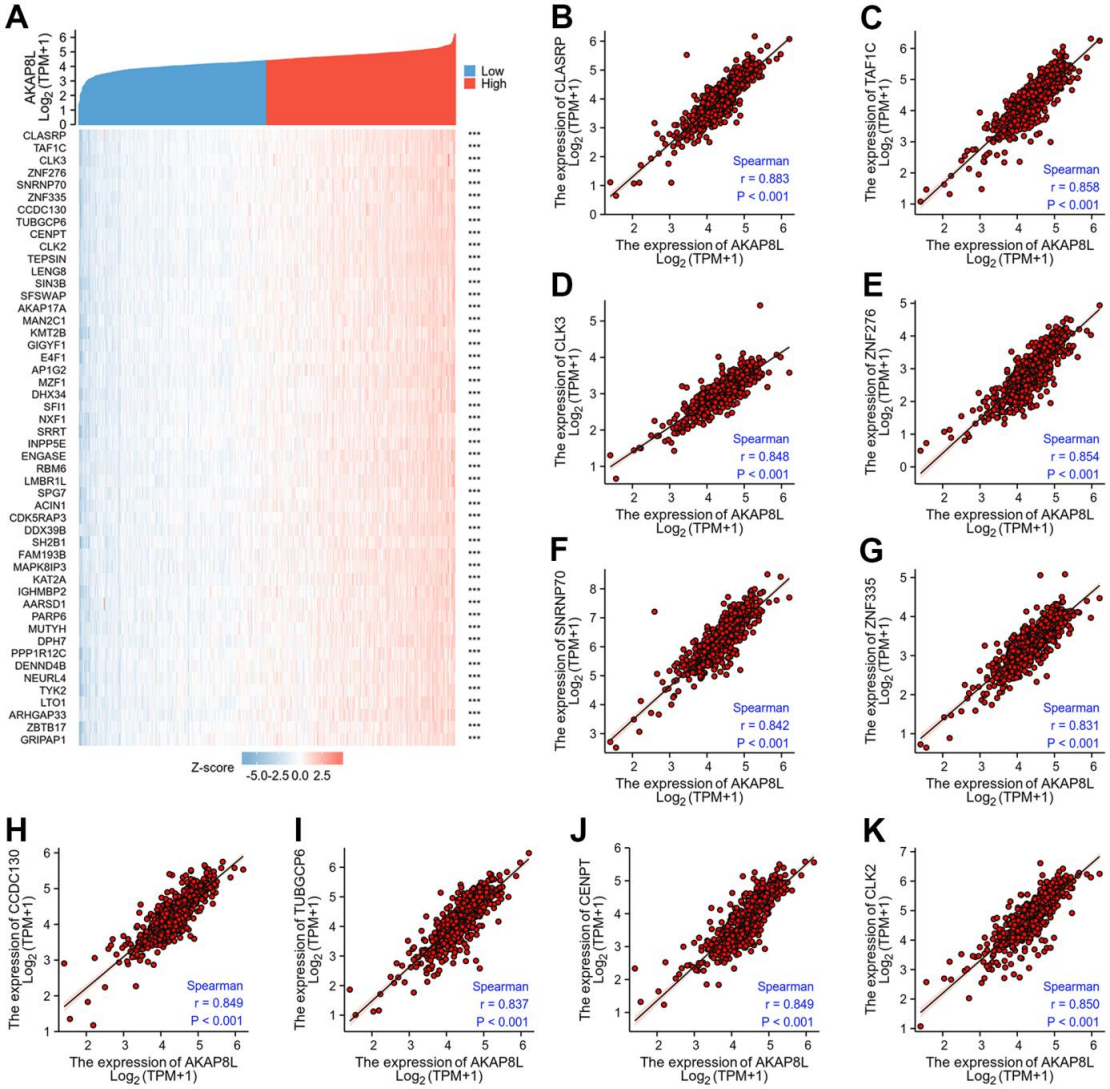
**Top 50 genes correlated with AKAP8L expression in KIRC.** (**A**) The gene co-expression heatmap of the top 50 genes correlated with AKAP8L in KIRC; (**B**–**K**) correlation analysis of the top 10 genes and AKAP8L in the heatmap.

The first 50 co-expression genes related to the expression of AKAP8L in KIRC, were investigated, and results showed the association of AKAP8L expression with the expression of the top 10 genes in the heat map, including CLASRP (r=0.883), TAF1C (r=0.858), CLK3 (r=0.848), ZNF276 (r=0.854), SNRNP70 (r=0.842), ZNF335 (r=0.831), CCDC130 (r=0.849), TUBGCP6 (r=0.837), CENPT (r=0.849), and CLK2 (r=0.850) (Figure 12A–12K).

### DEGs between AKAP8L high and low expression groups in KIRC

**Figure 13:**
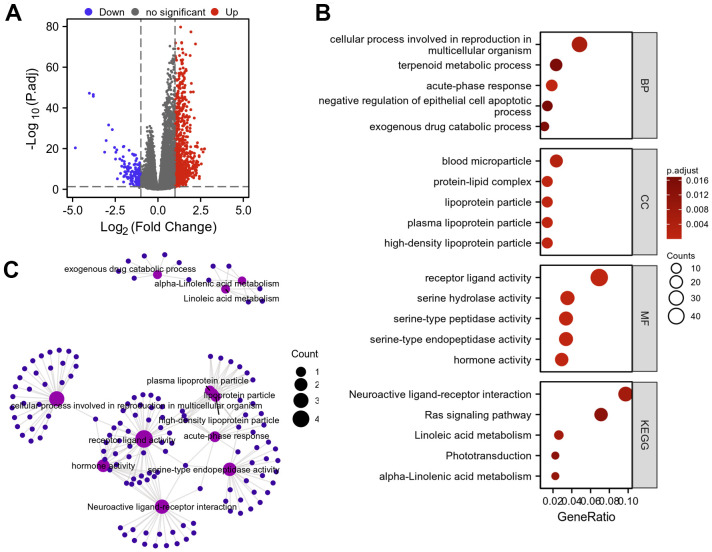
**Protein–protein interaction (PPI) network building and GO and KEGG analyses of DEGs between AKAP8L high expression and low expression groups in KIRC.** (**A**) The volcano map of DEGs (red: upregulation; blue: downregulation); (**B**, **C**) GO and KEGG analyses of DEGs.

In total, 855 DEGs were obtained with a threshold of | log2 fold-change (FC) |>1.0 and adjusted p value<0.05, among which 709 were up-regulated and 146 were down-regulated in KIRC compared to normal tissues (Figure 13A). Subsequently, GO and KEGG enrichment analysis were conducted on DEGs identified in KIRC (Figure 13B, 13C). Our analysis revealed that primary BP enriched by the DEGs included acute-phase response, cellular process involved in reproduction in multicellular organism, exogenous drug catabolic process, terpenoid metabolic process and negative regulation of epithelial cell apoptotic process. CC was primarily involved in high-density lipoprotein particle, plasma lipoprotein particle, lipoprotein particle, protein-lipid complex and blood microparticle. MF enrichment was mainly related to receptor ligand activity, serine-type endopeptidase activity, hormone activity, serine hydrolase activity and serine-type peptidase activity. KEGG pathway was mainly enriched in the interactions between neuroactive ligands and receptors, the metabolism of linoleic and alpha-Linolenic acids, Ras signaling pathway as well as phototransduction.

### Relationship of AKAP8L expression with tumor-infiltrating immune cells

**Figure 14:**
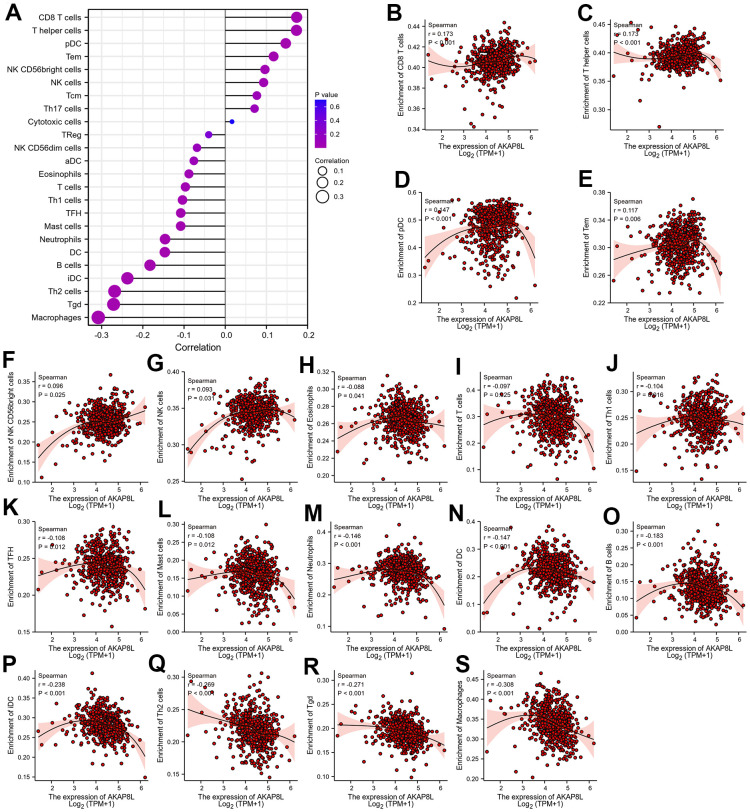
**Relationship between AKAP8L expression and tumor-infiltrating immune cells.** (**A**) The lollipop diagram of the correlation between AKAP8L expression and tumor-infiltrating immune cells. Relationship between AKAP8L expression and tumor-infiltrating immune cells, including (**B**) CD8 T cells, (**C**) T helper cells, and (**D**) pDC cells, (**E**) Tem, (**F**) CD56bright cells, (**G**) NK cells, (**H**) Eosinophils, (**I**) T cells, (**J**) Th1 cells, (**K**) TFH, (**L**) Mast cells, (**M**) Neutrophil, (**N**) DC, (**O**) B cells, (**P**) iDC, (**Q**) Th2 cells, (**R**) Tgd, (**S**) Macrophages.

Spearman correlation was adopted to investigate the relationship between the expression level of AKAP8L (TPM) and the level of GSEA quantitative immune cell infiltration in tumor microenvironment of KIRC (Figure 14A). We found that AKAP8L was pivotal in immune infiltration. AKAP8L was positively correlated with CD8 T cells, T helper cells, pDC cells, Tem cells, NK CD56bright cells, and NK cells (Figure 14B–14G), but negatively related to Eosinophils, T cells, Th1 cells, TFH cells, Mast cells, Neutrophils, DC cells, B cells, iDC cells, Th2 cells, Tgd cells, and Macrophages (Figure 14H–14S).

## DISCUSSION

AKAPs belong to various scaffold protein families, and these proteins are crucial in establishing the space of multivalent signal combinations [19, 20]. In particular, for g protein-coupled receptors, AKAPs serve as a central hub in tissues for G protein-coupled receptors, allowing for the assembly of multiple protein kinases and phosphatases to create signal devices able to send signals, regulate and transport within cells [21, 22]. It has been proved that AKAPs are pivotal for the initiation / progression of tumors and serve as potential targets of drug therapy [7, 23]. For example, Zhang et al. confirmed that AKAP4 was up-regulated in the tissues of non-small cell lung cancer, and the knockdown of AKAP4 inhibited tumor growth as well as epithelial-mesenchymal transformation, suggesting that AKAP4 might be a pivotal target in treating this disease [24]. As demonstrated by studies, AKAP8L stimulates cell proliferation / migration in various cancers, such a sd colon cancer, gastric cancer and esophageal squamous cell carcinoma, and can interact with mTORC1 and promote cell growth. Thus, it may be a prospective target for the treatment of cancers [9, 15, 16, 25].

Currently, no study has investigated the significance of AKAP8L in a pan-cancer context. To investigate AKAP8L’s expression level in pancancer, we examined the HPA database and TCGA database. Our analysis revealed that AKAP8L was significantly up-regulated in 13 human cancers, but down-regulated in KICH. AKAP8L has played the role of oncogene in most malignant tumors and may take part in the initiation and progression of tumors. In addition, the expression level of AKAP8L was related to the molecular subtypes of 9 kinds of tumors. For example, AKAP8L expression was the highest in the C1 molecular subtype of KIRP, the basal molecular subtype of HNSC, as well as the CIN molecular subtype of COAD. At the same time, AKAP8L was related to distinct immune subtypes of 7 kinds of tumors. Previous studies have proved that AKAP8L was linked to the molecular subtypes of breast cancer [13]. Thus, our study on different cancer molecular subtypes or immune subtypes may provide a suitable entry point for the exploration of the role of AKAP8L. Besides, we also conducted an evaluation of the methylation level of AKAP8L among the tissues of pancancer and found AKAP8L the promoter was hypermethylated in CHOL, KIRP, KIRC and LIHC, but hypomethylated in BLCA and LUSC.

Various studies have demonstrated that AKAPs can impact the prognosis of multiple types of cancers [26–28]. In our study, we investigated whether AKAP8L could serve as a valuable diagnostic and prognostic marker in pancancer using ROC curves and Kaplan - Meier survival curves. Our analysis revealed that AKAP8L has a certain accuracy in predicting 15 types of cancer, with particularly higher accuracy in the prediction of COAD, LIHC, READ and TGCT. In addition, AKAP8L was significantly related to the prognostic markers of COAD, KIRC, KIRP as well as PRAD. Based on the above findings, it could be inferred that AKAP8L may possess significant diagnostic and prognostic value in a number of different tumor types, making it a potentially promising target for precision oncology. Thus, AKAP8L was crucial in mediating signal transduction both inside and outside the cell.

Furthermore, our study focused on analyzing the specific role of AKAP8L in KIRC, and found that high AKAP8L expression may lead to worse OS, DSS or PFI in clinical subgroups of KIRC with an age greater than 80, white, hemoglobin low, or histologic grade in G3 or G4. Thus, our results comprehensively and deeply analyzed the role of AKAP8L in KIRC. In addition, we accurately explored the relationship between AKAP8L expression and distinct outcomes of clinical subgroups of KIRC.

Subsequently, we identified the top 10 co-expression genes of AKAP8L, including CLASRP, TAF1C, CLK3, ZNF276, SNRNP70, ZNF335, CCDC130, TUBGCP6, CENPT, and CLK2. In addition, we carried out GO and KEGG pathway analysis on DEGs between AKAP8L high and low expression group, and revealed that BP was mainly related to acute-phase response, cellular process involved in reproduction in multicellular organism, exogenous drug catabolic process, terpenoid metabolic process, negative regulation of epithelial cell apoptotic process as well as diterpenoid metabolic process. MF was mainly involved in receptor ligand activity, serine-type endopeptidase activity, hormone activity, serine hydrolase activity, growth factor activity along with substrate-specific channel activity. The main pathways were concentrated in interactions between neuroactive ligands and receptors, the metabolism of linoleic and alpha-Linolenic acids, Ras signaling pathway as well as phototransduction.

Analysis of genes involved in cancer development at the immune level may be able to increase our understanding of potential prognostic factors. Currently, many studies have proved a close relationship of immune infiltration with the prognosis and treatment response of various human cancers [29–31]. Studies have shown that AKAPs affect the immune infiltration and immunotherapeutic effects of several cancers [32–34]. Based on these facts, we conducted an analysis to examine the association between AKAP8L expression and the extent of immune cell infiltration in KIRC tumors, and proved the association of AKAP8L with the infiltration of numerous immune cells, including B cells, T cells, NK cells, Macrophages, Neutrophils, and Eosinophils, et al. Our results suggested that AKAP8L may influence the prognosis of patients with KIRC by mediating the level of immune cell infiltration in the microenvironment of KIRC.

Our research still had some limitations. We only used online database when exploring AKAP8L, and actual clinical data were unavailable. More research would be needed to verify our results in future.

In conclusion, AKAP8L may be related to the progression and prognosis of various tumors, especially KIRC. Our research provided a new dimension for a comprehensive understanding of the key role of AKAP8L in tumor progression, and a solid theoretical basis for the discovery of novel targets as well as prognostic markers for cancer treatment, with a particular emphasis on KIRC.

## MATERIALS AND METHODS

### The expression analysis of gene

We used HPA (https://www.proteinatlas.org) to analyze the expression of AKAP8L in normal tissues and in tumor cell lines [35]. We downloaded RNA-seq data and related clinical data of 33 tumor types and normal tissues of 10534 samples from the Cancer Genome Map (TCGA) database through UCSC XENA, used R software v3.6.3 for statistical analysis, and used ggplot2 package for visualization. Wilcoxon rank sum test was used to detect two groups of data, p< 0.05 is statistically significant (ns, p ≥ 0.05; *, p < 0.05;**, p < 0.01; ***, p < 0.001) [36].

### AKAP8L expression in molecular subtypes and immune subtypes of cancers

TISIDB database is used to explore the correlation between AKAP8L expression and molecular subtypes or immune subtypes in pan cancer [37]. The database integrates a variety of data used to evaluate the interaction between tumor and immune system.

### Protein–protein interaction network building

We obtained 50 AKAP8L binding proteins from STRING web, of which the parameters were set as the minimum required interaction score [medium confidence interval (0.400)] and active interaction source (“experiment, text mining, database”) [38, 39].

### Gene ontology and Kyoto encyclopedia of genes and genomes enrichment analyses

We used cluster profiler package for statistical analysis, ggplot2 package for visualization, and gene ontology (GO) and Kyoto Encyclopedia of Genes and Genomes (KEGG) enrichment analysis of 50 AKAP8L binding proteins [40].

### Diagnostic value analysis

The receiver operating characteristic (ROC) curve was used to evaluate the diagnostic value of AKAP8L in pan cancer. The closer the area under the curve (AUC) is to (1), the better the diagnostic effect is. AUC has a low accuracy in 0.5~0.7, a certain accuracy in 0.7~0.9, and a high accuracy above 0.9.

### Prognosis analysis

We used Kaplan-Meier plots to analyze the relationship between AKAP8L expression and tumor prognosis (OS, DSS, PFI). In addition, we further studied the relationship between AKAP8L expression and the prognosis of different clinical subtypes of KIRC (OS, DSS and PFI). We use survival package for statistical analysis and survivin package for visualization. We use Cox regression to test hypothesis, p< 0.05 is statistically significant [41].

### Patients

The tumor and adjacent normal tissues of 10 patients with confirmed KIRC were collected. This study was approved by the ethics committee of the First Affiliated Hospital of Nanchang University.

### Cell culture

Human kidney cell line (HK2) and human kidney cancer cell lines (A498 and 786-O) were obtained from American Type Culture Collection. Human kidney cell line (HK2) and human kidney cancer cell lines (A498) were cultured in MEM (minimum essential medium) media with supplements at 37° C with 5% CO_2_ in humidified air. Human kidney cancer cell lines (786-O) cultured in RPMI-1640 media with supplements at 37° C with 5% CO2 in humidified air.

### Reverse transcription-quantitative PCR (RT-qPCR)

Total RNA (from HK2, A498 and 786-O cells) was isolated using the total RNA extraction kit (Takara Bio, Inc). The Bestar™ qPCR RT Kit (Takara Bio, Inc) was used to synthesize cDNA from isolated total RNA. Samples were processed in the Applied Biosystems 7500 Real-Time PCR System using TB Green Premix Ex Taq II (cat. no. RR820A; Takara Bio, Inc). The reaction steps were as follows: i) Pre-denaturation, 95° C for 30 seconds; and ii) PCR reaction (40 cycles), 95° C for 5 seconds, 60° C for 30 seconds. The primer sequences for β-actin and AKAP8L are shown in Supplementary Table 1. Cycle threshold values were collected to calculate the relative expression of target genes.

### Tissue preparation and histopathological examination

Renal carcinoma and adjacent carcinoma samples were fixed in 10% formalin for 24 hours, then embedded in paraffin and cut into 3 μM thick sections for immunohistochemistry staining.

### Immunohistochemistry and immunofluorescence analysis

Paraffin sections were deparaffinized, rehydrated, immersed in antigen retrieval solution, and autoclaved at 121° C for 10 min for antigen retrieval. Sections were incubated with Serum-Free Protein Block (Dako) and pretreated with 100% methanol containing 3% hydrogen peroxide. Then, sections were incubated with the AKAP8L antibody (36068; Signalway Antibody). Primary-stained sections were incubated with appropriate peroxidase-conjugated secondary antibodies (GB23303; Servicebio) and diaminobenzidine substrate.

### Univariate and multivariate Cox regression analyses in KIRC

We used univariate and multivariate Cox regression to analyze AKAP8L and clinical features to determine its prognostic value in OS, DSS and PFI of KIRC. We use survival packages for statistical analysis.

### Co-expression gene analysis of AKAP8L in KIRC

We seeked the first 50 co-expressed genes related to AKAP8L expression in KIRC, and used stat package to display gene co-expression heatmap. We also used Pearson correlation coefficient to show the correlation between the first 10 genes and AKAP8L expression.

### DEGs between AKAP8L high expression and low expression groups in KIRC

We used the deseq2 package to study the DEG between different AKAP8L expression groups (low expression group: 0 – 50%; high expression group: 50 – 100%) in KIRC. We used ggplot2 package to draw the volcano map. The threshold value | log 2 times change (FC) |>1.0, and the adjusted p value<0.05.

### Promoter methylation analysis of AKAP8L

We used UALCAN (http://ualcan.path.uab.edu) to obtain the data on AKAP8L promoter methylation level in 33 tumor patients. Differences were compared by the Student’s t-test, and a p < 0.05 was set as statistically significant [17, 18].

### Relationship between AKAP8L expression and tumor-infiltrating immune cells

We used ssGSEA (single sample gene set enrichment analysis) method in GSVA package to analyze the immune infiltration of KIRC. And we quantified the relative enrichment fraction of each immune cell from the gene expression profile of each tumor sample based on the characteristic genes of 24 immune cells [42, 43]. We used Spearman correlation analysis to analyze the correlation between AKAP8L and these immune cells, and used Wilcoxon test to analyze the infiltration of immune cells between AKAP8L high expression group and low expression group.

### Statistical analysis

All collected data were analyzed using SPSS (IBM SPSS Statistics, version 20.0) and are expressed as the mean ± SEM. The difference between indicated groups was evaluated by Student’s t-test, Mann-Whitney U test, ANOVA or Kruskal-Wallis test. Student’s t-test (parametric) or Mann-Whitney U test (non-parametric) were used to compare the datasets of two groups. ANOVA (parametric) or Kruskal-Wallis test (non-parametric) were used to compare the datasets of multiple groups. P<0.05 was considered to indicate a statistically significant difference.

### Availability of data and materials

The datasets used and analyzed during the current study are available from the corresponding author on reasonable request.

## Supplementary Material

Supplementary Table 1
