# Supplementary material for: Integrative analysis identifies AKAP8L as an immunological and prognostic biomarker of pan-cancer
Source: Aging (Albany NY). 2023 Sep 7;15(17):8851–72. doi: 10.18632/aging.205003 (PMC10522372; doi:10.18632/aging.205003)
Supplement: Supplementary Table 1 [file aging-15-205003-s001.pdf]

SUPPLEMENTARY TABLE

Supplementary Table 1. Primer sequences of AKAP8L and β-actin.

Gene	Species	Forward	Reverse
β-actin	Human	5-TCTCCCAAGTCCACACAGG-3	5-GGCACGAAGGCTCATCA-3
AKAP8L	Human	5-AAACCGTGGAGGACCTTGATGG-3	5-AAGAGGTCGCAGGCTGCACAAT-3
